# Psychopathological aspects of sexting and IBSA perpetrators: A brief research report

**DOI:** 10.3389/fpsyt.2022.983881

**Published:** 2022-09-29

**Authors:** Aina M. Gassó, Carlos G. Forero, Jorge Piqueras, Esperanza L. Gómez-Durán

**Affiliations:** ^1^School of Medicine, Universitat Internacional de Catalunya, Barcelona, Spain; ^2^Galatea Clinic, Barcelona, Spain

**Keywords:** IBSA, perpetration, online sexual violence, psychopathology, intervention

## Abstract

**Introduction:**

IBSA has been defined as taking, distributing, and/or making threats to distribute, a sexual image without a person's consent, and up to date there is still limited research on IBSA perpetration and characteristics of IBSA perpetrators. Thus, the aim of this study was to identify characteristics of IBSA perpetrators, in order to guide future intervention and prevention programs.

**Methods:**

An online survey was conducted regarding IBSA related behaviors and psychopathology. The original sample comprised 1,370 Spanish college students (74% females).

**Results:**

The IBSA perpetrator subsample comprised 284 participants (49.5% females). Our findings indicate that perpetrators are more commonly males, with higher psychopathology scores, especially in hostility scales, with previous IBSA victimization experiences, and who usually target friends, to have fun or as a joke, or partners, to flirt. Furthermore, when examining intragroup differences regarding perpetration level of severity, results showed that those who reported engaging in the most severe forms of IBSA reported higher rates of psychopathology and hostility. Yet, to intervene in those who present more severe behaviors, we must also pay attention to depression, somatization and sleep disturbances.

**Conclusions:**

IBSA perpetrators share key factors that could be targeted in forensic and clinical interventions, and that should be taken into account when designing effective offender intervention programs. Intervention programs should focus on anger-management issues that help reduce perpetrators' hostility and anxiety symptoms, and should also be aimed at modifying attitudes that justify perpetration behaviors and contribute to harmful interactions with their friends or to intimate partner violent dynamics.

## Introduction

Due to the rapid development of technology, interpersonal communication, including intimate relationships and sexual interactions, have shifted to the online world (1, 2). The appearance of this new forms of communication have led to the development of new forms of contact, including the exchange of sexual content, also known as sexting (3, 4). Although there is no consensus around the definition of this phenomenon, sexting could be defined as the act of creating and sending nude or sexually explicit images or videos through any electronic device (3, 5, 6). This phenomenon has drawn increasing social and scientific attention, and research shows that it is a common practice both in adolescent populations, with approximately 12% of minors engaging in sexting, and in young adult populations, with almost 50% of adults reporting sexting engagement (6).

Sexting has been considered by some as a threshold for other forms of online victimization such as cyberbullying, online grooming or online sexual victimization (7–12). Again, although there is a lack of consensus around its definition, online sexual victimization has been defined by some authors as “*the experience of some of type of pressure through the Internet or mobile phones to obtain unwanted sexual contact or information (e.g., share sexual information, send images with sexual content, or do something against the victim's wishes) or/and the distribution or dissemination by the perpetrator of sexual images or sexual information of the victim against his/her will”* (9). This phenomenon has also been defined by other authors under the name of IBSA (Image based sexual abuse), as taking, distributing, and/or making of threats to distribute, a nude or sexual image without a person's consent (11, 13).

Englander (8) showed that 70% of her college student sample was pressured to sext, whilst Branch et al. (14) found that approximately 10% of their sample had been victims of revenge porn (having their intimate and sexual content disseminated without consent with the intention to get revenge). In the same line, Henry et al. (11) surveyed 4,274 Australian adults and reported that 1 out of 10 participants had sent sexual content to someone, and this content had then been disseminated without the person's consent. Furthermore, they found that 23% of their sample reported being victims of at least one form of IBSA, with the most common form of victimization being nude or sexual images being taken from them without their consent, reported by 20% of the sample (11). In Spain, being pressured to sext (28.2%), being pressured to share intimate or sexual information (24.5%), being pressured or threatened to perform a sexual act on the internet (22.2%), or being threatened online to maintain sexual intercourse with someone (18.7%) are more prevalent forms of victimization than the non-consensual dissemination of sexual content (4%) (9).

Although online sexual victimization or IBSA have been receiving increased attention, there is little evidence regarding IBSA perpetration and characteristics of perpetrators. Examining general online sexual behavior perpetration, in a sexting study of American adults aged between 21 and 75 years (*n* = 5.805), Garcia et al. (15) found that more than one in five participants (23%) reported sharing a “sexy” photo with someone else without consent. Another recent study carried out in Australia with 4053 participants showed that 11% of their sample had reported engaging in image-based sexual abuse perpetration (11, 16). Results indicated that men were significantly more likely to report IBSA perpetration than women. With regards to the nature of perpetration, participants reported targeting men and women at similar rates, and were more likely to report perpetrating against intimate partners or ex-partners, family members and friends than strangers or acquaintances (16). Findings also suggested that participants who had been victims of online sexual victimization were also more likely to report engagement in perpetration behaviors (16). Finally, a recent study carried out in Spain reported that 6.4% of participants had engaged in sexting coercion perpetration, with males being 7 times more likely to be perpetrators than females (17).

### Present study

Up to date there is still limited research on the extent and nature of IBSA perpetration, and characteristics of IBSA perpetrators, especially in Spain. However, based on previous literature, we hypothesized that males would report higher rates of sexting and IBSA perpetration, and higher prevalence rates of hostility. Thus, the aim of this study was to identify characteristics of IBSA perpetrators amongst a Spanish college sample, in order to guide future intervention and prevention programs.

## Methods

### Participants

The total sample recruited for this research comprised 1,370 Spanish college students (both undergraduate and post-graduate students, such as Master students), including 999 women (73.6%) and 359 men (26.2%). Ages ranged from 18 to 64 years old, with a mean age of 21.40 years (SD = 4.90). The subsample of sexting and IBSA perpetrators comprised 284 participants out of the total sample, with 49.5% females and 50.5% males.

### Instruments

#### Sexting and IBSA

We created a Sexting and IBSA Scale based on the JOV-Q (18) to assess different sexting, online sexual victimization and IBSA perpetration behaviors. For the purpose of this study, we assessed sexting and IBSA perpetration by asking participants to answer the following questions as described in the JOV-Q: Question 1 “I have forwarded to someone a photo/video of sexual content of myself”; Question 2: “I have forwarded to someone a photo/video of sexual content that I have received”; Question 3: “I have recorded or taken photos/videos of sexual content from someone else and I have sent it to a third person without consent”; Question 4: “I have pressured someone to send me photos/videos of their sexual content”; and Question 5: “I have threatened someone to send me photos/videos of their sexual content”.

On the other hand, we assessed victimization with the following items: (a) being a victim of non-consensual dissemination of nude or sexual images/videos of oneself, (b) being pressured to sext, and (c) being threatened to sext. The scale reported a Cronbach Alfa of 0.93 for online sexual victimization (19).

#### Psychopathology questionnaire

In order to measure mental health, we used the Spanish version of LSB-50, unlike other longer lists of symptoms such as the SCL-90-R, evaluates psychological and psychosomatic symptoms more quickly, with good reliability and validity parameters (20). This instrument consists of 50 items that assess psychopathological symptomatology. Responses to the items are collected on a 4-point Likert scale (0 = never and 4 = extremely). To analyze the presence or absence of mental health symptoms, the results obtained from the LSB-50 questionnaire were converted according to the authors guidelines (20). LSB-50 showed high reliability scores ranging from 0.82 to 0.90 reported by Abuín and de Rivera (20).

#### Perpetrated conduct

We considered a variable for general perpetration (anyone who had responded affirmatively to any of the 4 perpetration behaviors), and severity of perpetration (perpetration score) (21). This second variable is based on the results of a factor analysis on ordinal indicator based on IBSA perpetration items (0-Never done, 1- forwarded content of myself, 2- forwarded received content, 3- recorded and sent without consent, 4- pressured, 5-threatened), in an ordinal scale reflecting the severity of motivation for sharing the content (0- Never done; 1- flirting, 2- Joking, 3- Annoying the receiver; 4- Coercion and threatening) Participants were assigned to a single group according to the level of perpetration achieved, as if they were scales of perpetration severity.

### Procedures

The study was approved by the Ethics Committee of the International University of Catalunya (UIC Barcelona). Participation was voluntary and responses were anonymous to promote openness and honesty. The survey was administered online between March 2018 and June 2019. It included information about the nature and objectives of the study at the beginning of the questionnaire and informed consent was collected explicitly. The Survey link was sent to university professors from Spanish Universities with a request to pass it on to their students. The participating students then self-selected to take part in their own time, and no compensation was offered for participating. The questionnaire took approximately 20–25 min to complete, and once completed, students were given information on community resources in case of distress and the email address to contact the investigators in case of concerns. No participant contacted the investigators. The same online survey included all of the instruments mentioned in the previous section. This article is part of an ongoing work that uses the same sample as the one used in Gassó and Gómez-Durán (22) and Gassó et al. (17).

### Statistical analysis

Sample's sociodemographic characteristics, frequency of internet access and presence of psychological symptoms are presented in terms of percentage of respondents. We compared these variables between perpetrators and non-perpetrators using Chi-square Test and independent sample *T* tests.

Perpetration Scores were obtained applying a one-dimensional ordered categorical item response theory (IRT) model described by two parameter sets per item: a) one slope parameter per item, indicating amount of relationship between item and the severity trait and b) a number of threshold parameters equal to number of response categories minus 1, representing the score level needed to change from a less severe category to the following. Threshold parameters are on the same scale as the severity continuum and represent how extreme is the category in terms of severity (or how much severity is needed to choose a certain category and not the following). The model was estimated using an unweighted least squares estimator with a parameterization that produced item parameters in standardized normal IRT scale. Categories with too few responses for estimations were collapsed with the previous categories until the model was estimable. Good fit was stablished using root mean square error of approximation (RMSEA) less than 0.05 and Tucker Lewis index (TLI) over 0.90. Participant scores Individual were obtained as expected *a posteriori* estimates of the individual severity trait score under the model, scaled to a standard normal population distribution of mean 0 and standard deviation 1, with higher scores indicating higher perpetration severity score.

Differences in Perpetrator severity score and groups based on external variables was computed using independent sample *T*-tests for dichotomous variables, and for variables with more than 2 categories (either ordinal or nominal), Oneway ANOVA of variance or Kruskal-Wallis H depending on the fulfillment of minimum group sample sizes. In case a category had 2 or less responses, it was collapsed with the immediately inferior categories until a sample size for the collapsed category of 5 or more.

We used nominal alpha level α = 0.05 for decisions. Data was analyzed using SPSS v26 and MPlus 8.5 (23).

## Results

Our results showed that 20.7% of the sample reported engaging in at least one IBSA perpetration behavior in the previous year. Out of the group of participants who reported engaging in IBSA perpetration behaviors (*n* = 284), 63.4% forwarded sexual content they had received, 6% took a sexual picture of a victim and forwarded it without consent, 23.9% pressured someone to receive sexual content and 6.7% threatened someone to receive sexual content. Results from preliminary analysis showed differences between IBSA perpetrators and non-perpetrators, especially regarding some psychopathology measures and victimization rates. Results can be found in Tables 1, 2.

**Table 1:** Descriptive statistics of demographic and background variables for perpetrators and non-perpetrators.

	**Total sample % (*N =* 1,370)**	**Perpetrators** **(*N =* 284)**	**Non-perpetrtors (*N =* 1,045)**	**Chi-square or T-Student significance (*P*-value)**
Demographic variables				
**Gender**				
Male	26.40	50.5	19.3	0.000
Female	73.60	49.5	80.7	
**Age**	21.43 (SD 4.85)	21.46 (SD 4.36)	21.35 (SD 4.80)	0.720
**Marital status**				
Single	54.60	51.8	55.5	0.782
In relationship	42.00	45.10	41.5	
Married	1.20	1.10	1.10	
Common Law Partner	1.30	1.10	1.20	
Divorced/separated	0.90	1.10	0.70	
**Academic situation**				
Undergraduate	92.40	93.3	92.3	0.774
Master's Degree	4.00	3.50	3.90	
Erasmus	1.50	1.80	1.40	
Other	2.20	1.40	2.30	
**Living situation**				
With parents	62.40	62.4	62.4	0.734
Student apartment	22.40	25.10	22.10	
Off campus student residence	4.60	3.60	4.80	
On campus student residence	0.70	0.70	0.80	
Alone	3.80	2.50	3.90	
With partner	6.20	5.70	6.10	
**Employment status**				
Unemployed	67.40	67.60	67.40	0.812
Employed full time	5.10	3.90	5.00	
Employed partial time	27.40	28.50	27.50	
**Frequency of internet access**				
Once a week	0.10	0.40	0.20	0.655
2–3 times a week	0.40	0	0.10	
Everyday	33.0	28.10	33.60	
2–3 h per day	16.7	18.0	16.60	
More than 3 h per day	48.0	51.40	47.30	

**Table 2:** Psychopathology, victimization and perceived risk variables for perpetrators and non-perpetrators.

	**Total sample % (*N =* 1,370)**	**Perpetrators (*N =* 284)**	**Non-perpetrators (*N =* 1,045)**	**Significance (*P*-value)**
**Psychopathology**				
IGS	31.9	45.3	38.2	0.032
Psychoreactivity	50.8	52.5	50.1	0.472
Hypersensitivity	41.8	42.4	41.4	0.768
Obsessive-compulsive	50.9	56.2	49.6	0.053
Anxiety	49.5	50.4	49.3	0.758
Hostility	35.3	39.1	34.2	0.126
Somatization	28.3	31.9	27.2	0.129
Depression	29.9	35.5	28.0	0.016
Sleep alteration	26.8	30.8	25.7	0.208
**Victimization**				
Being victim of NCDS	3.3	4.30	3.0	0.292
Being a victim of pressure	32.7	41.0	30.4	0.001
Being a victim of threats	3.4	3.9	3.3	0.619
**Perceived risk**				
Risk of forwarding sexual content	13.2	37.7	4.0	0.000
Risk of taking and sending sexual content without consent	8.2	13.1	6.7	0.004
Risk of pressuring someone for sexual content	5.5	13.8	2.7	0.000
Risk of threatening someone for sexual content	2.7	3.0	2.6	0.733

When examining who the perpetrators confirmed having victimized: they mainly forwarded content of friends (78.5%) and strangers (14.6%), they were more likely to create and disseminate their friends' content (64.7%) followed by internet acquaintances' (23.5%); but they pressured partners (47.5%) followed by friends more frequently (24.6%); and finally they also threatened their partners (57.1%) followed by their friends (21.4%). Regarding the reasons why they stated perpetrating IBSA, participants reported that they forwarded someone else's content majorly as a joke or to have fun (96.1%); in the same line, they created and disseminated someone else's sexual content for fun or as a joke (82.4%); whilst they reported having pressured someone to send their sexual content to flirt (55.7%) followed by for fun (41.0%); and, similarly, they stated threating someone to send their sexual content to flirt (46.2%) and for fun (46.2%).

The model for the perpetration severity score showed excellent fit (RMSEA = 0.034; TLI = 0.94). The following Table 3 shows item parameters. Item 4 was the most related with perpetration severity (due to its high slope parameter/factor loading 0.85), while the item 1 was the least related (slope = 0.80, factor loading = 0.85). As for the location in the perpetration severity continuum, category thresholds were well ordered in the continuum from “never done” to “Coercion”. Certain item categories were too infrequent in the sample so that item parameters could not be estimated. In fact, pass to coercion category was only estimable for item 2. In terms of perpetration severity, item 4 and 5 implied the most extreme conducts (average threshold 8.6 and 6.06) while item 1 and 3 were the least severe (average threshold 2.61 and 1.93, respectively).

**Table 3:** Item parameters for the perpetration severity score model.

**Item**	**Slope (SE) /factor loading equivalent value**	**Threshold (SE)**			
		**1** **Never done**	**2** **Flirting**	**3** **Joking**	**4 Coercion**
1. Forwarded someone a photo/video of sexual content of myself	0.80 (0.29)/0.34	0.76 (0.29)	2.18 (0.20)	4.91 (0.39)	–^1^
2. Forwarded someone a photo/video of sexual content that I have received	1.33 (0.50)/0.66	4.85 (0.56)	4.94 (0.57)	7.35 (0.69)	8.06 (0.69)
3. Recorded or taken photos/videos of sexual content from someone else and I have sent it to a third person without consent	0.64 (0.17)/0.46	1.91 (0.11)	1.95 (0.11)	–^1^	–^1^
4. Pressured someone to send me their sexual content	5.80 (1.20)/0.85	4.43 (2.4)	7.68 (4.7)	13.7 (3.9)	–^1^
5. Threatened someone to send me their sexual content	1.44 (0.38)/0.52	5.7 (0.59)	6.42 (0,74)	–^1^	–^1^

1 Response category collapsed with the previous category.

Furthermore, the perpetration severity score was statistically related with several psychopathological variables (see Table 4).

**Table 4:** Perpetration severity score model differences in psychopathology.

**IGS**	**No**	**788**	**−0,01**	**0,02**	**0,000**
	**Yes**	**524**	**0,06**	**0,02**	
**IIS**	**No**	**1,112**	**−0,01**	**0,01**	**0,000**
	**Yes**	**200**	**0,15**	**0,04**	
Psychoreactivity	No	647	0,01	0,02	0,374
	Yes	665	0,02	0,02	
Hypersensitivity	No	764	0,00	0,02	0,333
	Yes	548	0,03	0,02	
Obsessive- Compulsive	No	643	0,00	0,02	0,427
	Yes	669	0,03	0,02	
Anxiety	No	662	0,00	0,02	0,178
	Yes	650	0,03	0,02	
**Hostility**	**No**	**849**	**−0,02**	**0,01**	**0,001**
	**Yes**	**463**	**0,08**	**0,02**	
**Somatization**	**No**	**939**	**−0,01**	**0,01**	**0,000**
	**Yes**	**373**	**0,08**	**0,03**	
**Depression**	**No**	**922**	**−0,02**	**0,01**	**0,000**
	**Yes**	**390**	**0,09**	**0,03**	
**Sleep alteration**	**No**	**959**	**0,00**	**0,01**	**0,000**
	**Yes**	**352**	**0,07**	**0,03**	
**Sleep alteration amplified**	**No**	**900**	**0,00**	**0,02**	**0,007**
	**Yes**	**411**	**0,06**	**0,03**	
**IRP**	**No**	**690**	**−0,01**	**0,02**	**0,008**
	**Yes**	**621**	**0,04**	**0,02**	

IGS, Global Severity Index; IIS, Intensity of Symptoms; IRP, Psychopathological Risk Index. Bold values represent significant values.

## Discussion

Overall, our results showed that 20.7% of the original sample reported engaging in at least one IBSA perpetration behavior in the previous year, which is higher than the results obtained in previous research, with rates that range between 5.1 and 35% (11, 15, 24–28). Out of the group of participants who reported engaging in IBSA perpetration behaviors, 63.4% forwarded sexual content they had received, 6% took a sexual picture of a victim and forwarded it without consent, 23.9% pressured someone to receive sexual content and 6.7% threatened someone to receive sexual content. Henry et al. (11) study showed that behaviors involving the taking of a nude or sexual image (8.7%) were the most common, followed by behaviors involving the distribution of a nude or sexual image (6.4%), and behaviors involving threats to distribute a nude or sexual image (4.9%). Differences in the reported measures could be due to cultural differences (28, 29), but also to differences in conceptualization of the measured behaviors, samples and instruments used (11).

Our data shows gender differences between groups, with males being 2.9 times more likely to engage in IBSA perpetration than females. These results are in line with previous research (11, 15, 16, 24, 26, 28). Other demographic variables showed slight differences between IBSA perpetrators and non-perpetrators. Most background variables did not vary between both groups, such as marital status, academic situation, living situation, employment status or frequency of internet access, in line with previous research (11, 17, 22, 29). However, results showed significant differences for global psychopathology, with perpetrators showing higher rates of psychopathology than non-perpetrators. Despite the fact that, to our knowledge, no studies have examined psychopathology of IBSA perpetrators, Clancy et al. (30), Clancy et al. (27) found significant associations between disseminating sexts and traits such as psychopathy, machiavellianism and narcissism. Furthermore, Gassó and Gómez-Durán (22) measured psychopathology in a sample of sexting coercion perpetrators, and found higher psychopathology scores in all of the measured items, with results showing significant differences in the mean scores of psychoreactivity, hypersensitivity, hostility, somatization and depression.

It has been previously suggested that the type, form, or context of sexting and IBSA are especially important in considering whether and how they are related to psychosocial health (31). Accordingly, when comparing perpetrators by level of severity, our results showed that those who reported engaging in the most severe form of IBSA reported higher rates of global psychopathology, hypersensitivity and anxiety, but more obviously hostility. Hostility, as measured by the LSB-50, evaluates the presence of reactions of loss of emotional control with sudden or continuous manifestations of aggressiveness, anger, rage or resentment (20). This research finding suggests that interventions that focus on strategies for managing anger and aggressive impulses could have a role in preventing IBSA perpetrating behaviors. Yet, according to our severity score, to intervene in those who present more severe behaviors, both because of the behavior itself and because of its motivation, attention should also be paid to affective psychopathology. Results suggest that depression, somatization and sleep problems, in addition to hostility could be especially relevant and would need to be specifically addressed.

Furthermore, the low percentage of perpetrators who perceived IBSA behaviors as risky could also represent a possibility of intervention. Previous literature has stated the association between IBSA perpetration and negative attitudinal trends toward sexual violence, such as victim blaming, while also minimizing, excusing or justifying their perpetrator behaviors (28, 32, 33). Yet, this finding should also be linked with motives for IBSA perpetration reported by our participants. Some research has stated that perpetrators may engage in euphemistic labeling, or, in other words, claim they engaged in IBSA perpetration to be funny, or to make a joke or a prank, which, according to this study, could potentially be a commonly used mechanism of moral disengagement in the context of IBSA (34). A recent study found that approximately 31% of the participants who had disseminated a sext without consent did so as a joke, with male participants being significantly more likely to endorse this excuse (27, 30). Our results are in line with those obtained by Clancy et al. (30), Clancy et al. (27), with “for fun” or “as a joke” being the most common self-reported motives for engaging in IBSA perpetration. Our results highlight the need for deeper research in intervention of these attitudes.

Moreover, our results are consistent with previous research that addresses both IBSA perpetration and victimization and suggests both experiences are strongly related, with participants who self-report perpetration more likely to report victimization in their lifetime (16, 27, 30, 35). Preventing IBSA victimization experiences and intervening early with victims could potentially contribute to IBSA perpetrating behaviors prevention.

Finally, when examining relational patterns of IBSA perpetration, several studies have further found that IBSA images were most commonly shared with close friends or other friends (26, 35), in line with our results. Our data showed that perpetrators reported victimizing friends and partners more commonly, which suggests that, similarly to offline sexual violence, it is more likely to occur within pre-established relations, rather than with strangers (36). According to our results, pre-established relationships between victim and perpetrator were more frequent among the higher levels of IBSA perpetrating behaviors. Therefore, our results suggest that IBSA related behaviors could be included in intimate partner violence offender intervention programs.

In accordance with our results and given the importance and complex nature of IBSA behaviors, prevention and intervention strategies with offenders need to be targeted at different levels and data provided hereby point to the potential benefits of integrating certain issues that could lead to more substantial gains in offender programs.

This study has several limitations that should be considered when interpreting the results. First, the sample used was non-probabilistic and relied on self-reported data, and the sample was composed of only university students, rather than the general population, so generalization of results should be cautiously done. In this sense, the sample used was self-selected using an online survey, which would explain why the total sample is unbalanced regarding female and male participants. Additionally, sexting and IBSA perpetration were measured by direct questions, which can create defensivity and rejection to answer the question with openness and honesty. Finally, this study is a cross-sectional investigation, and not longitudinal, so no causality can be established between the examined variables.

## Conclusions

Image based Sexual abuse, also known as IBSA, is a form of online sexual violence, which has increased over the past few years, with little evidence regarding the extent, nature and characteristics of IBSA perpetrators. To our knowledge, this is the first study to examine IBSA perpetrator characteristics and psychopathology amongst a Spanish college sample, in order to guide future forensic and clinical interventions. Our results indicate that IBSA perpetrators could share key factors that could be targeted in forensic and clinical interventions, and that should be taken into account when designing effective offender intervention programs. In this sense, results showed that perpetrators are more commonly male, with higher psychopathology scores, especially in anxiety and hostility scales, with previous IBSA victimization experiences, and who usually target friends, to have fun or as a joke, or partners, to flirt. These characteristics indicate that intervention programs should eventually be focused on anger-management issues that help reduce perpetrators' hostility and anxiety symptoms. Furthermore, our results highlight the importance of interventions aimed at modifying attitudes that justify these behaviors and contribute to harmful interactions with their friends or to intimate partner violent dynamics. Finally, another key factor in IBSA perpetration is that a high number of perpetrators have also been victims of IBSA, which means that reducing IBSA victimization might also contribute to reduce IBSA perpetration. Overall, this study has shown that IBSA perpetration is a complex behavior, that needs to be targeted at different levels and requires extensive research, and future investigations should further examine the relationship between IBSA perpetration and other relevant factors such as violent pornography consumption.

## Data availability statement

The original contributions presented in the study are included in the article/supplementary material, further inquiries can be directed to the corresponding author/s.

## Ethics statement

The studies involving human participants were reviewed and approved by Ethics Committee of the International University of Catalunya (UIC Barcelona). The patients/participants provided their written informed consent to participate in this study.

## Author contributions

AG and EG-D: conceptualization. AG, EG-D, and CF: methodology. AG, EG-D, CF, and JP: formal analysis. AG: writing—original draft preparation and formatting. EG-D and CF: writing—review and editing. All authors have read and agreed to the published version of the manuscript.

## Funding

This research has been conducted as part of the project Sexting victimization during the COVID-19 pandemic: Toward an evidence-based educational paradigm and financed by the Fundación MAPFRE as part of the Ignacio Hernando de Larramendi Grants (2020). Grant No. 6515.

## Conflict of interest

The authors declare that the research was conducted in the absence of any commercial or financial relationships that could be construed as a potential conflict of interest.

## Publisher's note

All claims expressed in this article are solely those of the authors and do not necessarily represent those of their affiliated organizations, or those of the publisher, the editors and the reviewers. Any product that may be evaluated in this article, or claim that may be made by its manufacturer, is not guaranteed or endorsed by the publisher.
